# Central adiposity increases the odds for plasma folate deficiency among Chinese women of reproductive age

**DOI:** 10.3389/fpubh.2022.1000542

**Published:** 2022-10-14

**Authors:** Jinjuan Zhang, Yushan Du, Xiaoyu Che, Shuangbo Xia, Le Zhang, Jufen Liu

**Affiliations:** ^1^Department of Critical Care Medicine, Peking University People's Hospital, Beijing, China; ^2^Institute of Reproductive and Child Health/National Health Commission Key Laboratory of Reproductive Health, Peking University, Beijing, China; ^3^Department of Epidemiology and Biostatistics, School of Public Health, Peking University, Beijing, China

**Keywords:** folate deficiency, central adiposity, waist circumference, body mass index, general adiposity

## Abstract

**Objective:**

To explore the association between adiposity and plasma folate deficiency odds among women of reproductive age in China.

**Methods:**

A cross-sectional survey on nutritional status among women of reproductive aged 18–30 years in 2005–2006 in China was conducted. General adiposity was defined as body mass index (BMI) ≥24 kg/m^2^, and central adiposity was defined as waist circumference >80 cm. A plasma folate concentration <10.5 nmol/L (measured through microbiological assay) was defined as plasma folate deficiency. Odds ratios (ORs) and 95% confidence intervals (CIs) for plasma folate deficiency were calculated using a logistic regression model, with adjustment for potential confounders.

**Results:**

A total of 3,076 women of reproductive age were included in the final analysis. Compared to women with normal BMI and WC, women with both general and central adiposity had the highest odds for plasma folate deficiency (OR = 3.107, 95% CI: 1.819–5.307). Women with exclusively central adiposity had excess odds for plasma folate deficiency (WC > 80 cm, BMI <24 kg/m^2^; OR = 2.448, 95% CI: 1.144–5.241), which was higher than women with exclusively general adiposity (BMI ≥ 24 kg/m^2^, WC ≤ 80 cm; OR = 1.709, 95% CI: 1.259–2.319). The combined use of BMI and WC can detect more women (11.7%) at higher plasma folate deficiency odds than either used alone.

**Conclusions:**

Women with central adiposity in normal weight have higher odds for plasma folate deficiency than those with general obesity only. Early screening for central adiposity among women of reproductive age would be meaningful to prevent folate deficiency and improve life-cycle health.

## Introduction

Emerging evidence has suggested that folate deficiency during the periconceptional period can result in a variety of negative health outcomes, both for mothers (e.g., megaloblastic anemia) and their offspring (e.g., neural tube defects and other congenital malformations) (1, 2). Excess accumulation and storage of body fat may affect folate absorption and metabolic pathways in the body (3, 4). Body mass index (BMI), a widely used measure of excess body weight, was found to be correlated with blood folate status, but the conclusions remain controversial. Epidemiology studies have revealed that increasing BMI was associated with lower serum folate concentration and/or higher red blood cell (RBC) folate levels (5–7), but some studies failed to find this relationship (8, 9). A hypothesized mechanism for this inconsistency was that folate status may differ widely between tissues, and obesity types (central adiposity or general adiposity) may have different effects on intracellular folate metabolism in different tissues (10). The use of BMI is inadequate if the fat distribution can affect folate levels because BMI was not available to reflect the body shape.

Visceral adipose tissue (VAT) is one of the most deleterious fat deposits in the body, and central adiposity due to VAT has a higher risk for cardiovascular diseases, metabolic disorders and cancers than general adiposity (11, 12). VAT cannot be detected by BMI, and waist circumference (WC), waist-to-hip ratio (WHR) and waist-to-height ratio (WHtR) are known indexes that reflect central adiposity, among them WC is an easily measurable surrogate index of abdominal fatness. WC has also been shown to be associated with folate status among postmenopausal women (13), school children (14), hypertensive adults (15), and other populations (16), but few of these studies targeted women of reproductive age.

The results from previous studies reported that central adiposity may be more strongly tied to metabolic risks than general adiposity, mainly indicated by BMI. Thus, it is reasonably postulated that central adiposity would be positively correlated with a higher risk for plasma folate deficiency than general adiposity, but few studies have examined this hypothesis. In addition, body adiposity based on BMI may underestimate adiposity among non-Caucasian populations, especially Asian populations, including Chinese populations (17, 18). It was reported that the combination of BMI and measures of central adiposity, such as WC, may be more effective in distinguishing subjects at risk than either alone (19). BMI and WC combined can better classify the subgroup of individuals with normal weight but with metabolic disturbances who present with excessive VAT, which would be neglected due to the desired BMI range (20). Evidence on adiposity defined by BMI and WC combination and blood folate status among women of reproductive age has not been widely investigated.

In the current study, we aimed to explore the association between adiposity (defined by BMI, WC, and the combination of BMI and WC) and plasma folate deficiency and whether BMI and WC combined was better for identifying high-odds subgroups for plasma folate deficiency than either alone among women of reproductive age aged 18–30 years in China.

## Materials and methods

### Study design and study population

The original study was conducted as a nationwide cross-sectional study that aimed to investigate the nutritional status of women aged 18–30 years in multiple regions in China, as reported elsewhere (21). Briefly, one community or village as a project site in each province during two periods (April to May 2005 and October 2005 to April 2006) was selected, and 100 women were planned to be recruited at each site. The inclusion criteria were as follows: (1) age 18–30 years; (2) no pregnancy or breastfeeding; (3) living in the area for more than 1 year and (4) free from hypertension, diabetes, cancers, and heart, liver, renal and gastrointestinal diseases.

A total of 3,660 women were recruited. We excluded 584 women who were out of age (5.55%), had serious diseases (5.14%), and missing data on plasma folate concentration (5.32%), BMI (0.24%) and waist circumference or hip circumference (0.34%). A total of 3,076 targeted participants (94.14% of eligible women) were included in the final analysis.

### Assessment of sociodemographic and personal characteristics

After obtaining consent, a face-to-face interview was conducted by trained healthcare workers with a structured questionnaire. Information on sociodemographic details and personal characteristics, including age, ethnicity, educational levels, current cigarette and alcohol use, use of oral contraception, and use of folic acid supplements or multivitamins containing folic acid (in the past 3 months), was collected. Dietary data were obtained using a 24-h recall through a face-to-face interview by trained nurses, with two-dimensional drawings of bowls and plates to estimate portion sizes. Dietary folate levels were calculated based on the Chinese Food Composition (22). Dietary folate intake (μg) was adjusted by energy intake to minimize the influence of false dietary reporting, calculated through 1,000^*^dietary folate intake (μg)/energy intake (kcal). Dietary folate adjusted by energy was then divided into three groups according to the tertiles of its natural logarithm (low: ≤1.98, medium: 1.98–2.16, and high: ≥2.16).

### Measurements of anthropometric parameters and definitions

Body weight in light clothing and height without shoes were measured to the nearest 0.1 kg and 0.1 cm with a beam weighing scale and a height scale, respectively. WC was measured with the subject standing at the level midway between the lower rib margin and the iliac crest, and hip circumference was measured at the level of the greater trochanters.

General adiposity was measured by BMI, which was calculated as weight (kg)/height (m)^2^) and categorized as underweight (≤18.5 kg/m^2^), normal weight (18.5–23.9 kg/m^2^), and overweight (≥24.0 kg/m^2^) according to the guidelines for Prevention and Control of Overweight and Obesity in Chinese Adults (23). Obesity cases (*n* = 82) were combined with overweight cases due to limited sample size. Central adiposity in the current study was defined as WC higher than 80 cm (24). The combination of BMI and WC categories was divided into 5 groups based on their BMI and WC values: (1) non-increased BMI and WC (BMI ≤ 18.5 kg/m^2^; WC ≤ 80 cm), (2) normal BMI and WC (BMI: 18.5–24; WC ≤ 80 cm), (3) exclusively central adiposity (BMI, 18.5–24; WC > 80 cm), (4) exclusively general adiposity (BMI ≥ 24.0 kg/m^2^, WC ≤ 80 cm), and (5) both central and general adiposity (BMI ≥ 24.0 kg/m and WC > 80 cm). Both waist-to-hip ratio (WHR) and waist-to-height ratio (WHtR) are ratio measures reflecting visceral fat mass and related to various health outcomes. Whether WHR or WHtR is associated with folate status is unknown in previous studies. So we also included them in our analysis. The cutoff values of abnormal WHR and WHtR were 0.85 and 0.5, respectively (25, 26).

### Blood sampling and laboratory analysis

Venous blood (9 ml) was collected after an overnight fast. Blood samples were drawn into potassium ethylene diamine tetraacetic acid (K3EDTA)-containing Vacutainer tubes (Becton Dickinson) and centrifuged within 1 h of collection. Plasma and erythrocytes were separated and frozen at −20°C. All specimens were transported on dry ice to the central laboratory of the Institute of Reproductive and Child Health, Peking University, and stored at −70°C before nutritional analyses. Plasma folate concentrations were determined by a microbiological assay (27). Plasma folate deficiency was defined as <10.5 nmol/L (28). The intra- and interassay coefficients of variation were both <9% across the full range of folate concentrations.

### Statistical analysis

Categorical variables are presented as frequencies and percentages. A chi-square test was adopted to examine differences in plasma folate deficiency (<10.5 nmol/L vs. 10.5 nmol/L) in each category. Binary logistic regression was performed to examine the association between general adiposity (measured by BMI) and central adiposity (measured by WC) and plasma folate deficiency odds while controlling for potential confounders, including sociodemographic factors [age (18–24, 25–30 years), education (junior high school or lower, high school and secondary school, college or higher), occupation (farmers, workers, unemployed, others), ethnicity (Han ethnicity, others), residence type (urban, rural), season of enrollment (spring, autumn), region of residence (north, south), income (<3,000 Yuan per year, 3,000–7,000 Yuan per year, ≥7,000 Yuan per year)], lifestyle characteristics [smoking (no, yes), drinking (no, yes), FA supplement (no, yes), use of oral contraceptives (no, yes), delivery status (none, previous)] and dietary intake (intake of dietary folate adjusted by energy (low, medium, high)] and WHtR (≤0.85, >0.85). Further analysis was performed to test the association between exclusively general adiposity and exclusively central adiposity (measured by the combination of BMI and WC) and plasma folate deficiency odds after adjusting for the same potential confounders except for BMI and WC. The Little's MCAR test was performed to test whether the missing data were totally at random. Considering the missing values in the multivariate logistic model, imputation method was used. Missing data with normal distribution was replaced by mean and skewed distribution by median. For category variables, we substituted the missing values with the majority type of the variable. Two-tailed *P*-values of < 0.05 were considered statistically significant. The analyses were performed using SPSS for Mac software version 26.0 (IBM, Chicago, IL, USA).

## Results

### Basic characteristics

The Little's MCAR test showed that the missingness was entirely at random (χ^2^ =1.157, *P* = 0.282). Among the 3,076 women of reproductive age, the overall plasma folate deficiency rate was 29.8%. The mean (SD) BMI and WC were 21.37 (4.17) kg/m^2^ and 70.05 (7.79) cm, respectively. The mean (SD) WHR and WHtR were 0.77 (0.06) and 0.44 (0.05). A total of 61.7% of women were aged 25–30 years, and 67.7% of them were of Han ethnicity. Almost half of the participants (47.2%) finished junior high school or lower. Women who were farmers and workers accounted for 31.4 and 11.7%, respectively. Only 4.7% of women reported having previously taken folic acid supplements. Smoking and drinking accounted for 1.7 and 8.0%, respectively (Table 1).

**Table 1:** Basic characteristics among Chinese women aged 18–30 years, 2005–2006^*^^#^.

**Characteristics**	**Total**	**Plasma folate**	**Non-plasma folate**	***P***
	**(*n* = 3,076)**	**deficiency (*n* = 918)**	**deficiency (*n* = 2,158)**	
**Age (years)**				
18–24	1,177 (38.3)	390 (42.5)	787 (36.5)	0.002
25–30	1,899 (61.7)	528 (57.5)	1,371 (63.5)	
**Ethnicity**				
Han ethnic	2,083 (67.7)	486 (52.9)	1,597 (74.0)	< 0.001
Others	993 (32.3)	432 (47.1)	561 (26.0)	
**Education**				
Junior high school or lower	1,452 (47.2)	428 (46.6)	1,024 (47.4)	0.588
High school and secondary school	833 (27.1)	260 (28.3)	573 (26.6)	
College or higher	791 (25.7)	230 (25.1)	561 (26.0)	
**Occupation**				
Farmers	966 (31.4)	201 (21.9)	765 (35.4)	< 0.001
Workers	360 (11.7)	134 (14.6)	226 (10.5)	
Unemployed	312 (10.1)	110 (12.0)	202 (9.4)	
Others	1,438 (46.8)	473 (51.5)	965 (44.7)	
**Income [Yuan (USD** ^$^ **), per year]**				
<3,000 (442.8)	1,090 (35.4)	296 (32.2)	794 (36.8)	< 0.001
3,000–7,000 (442.8–1033.2)	972 (31.6)	365 (39.8)	607 (28.1)	
≥7,000 (1033.2)	1,014 (33.0)	257 (28.0)	757 (35.1)	
**Residence type**				
Urban	1,525 (49.6)	395 (43.0)	1,130 (52.4)	< 0.001
Rural	1,551 (50.4)	523 (57.0)	1,028 (47.6)	
**Oral contraceptive**				
No	3,020 (98.2)	892 (97.2)	2,128 (98.6)	0.006
Yes	56 (1.8)	26 (2.8)	30 (1.4)	
**Smoking**				
No	3,025 (98.3)	898 (97.8)	2,127 (98.6)	0.140
Yes	51 (1.7)	20 (2.2)	31 (1.4)	
**Drinking alcohol beverage**				
No	2,831 (92.0)	823 (89.7)	2,008 (93.0)	0.001
Yes	245 (8.0)	2,008 (10.3)	150 (7.0)	
**Folic acid supplementation**				
No	2,930 (95.3)	890 (96.9)	2,040 (94.5)	0.004
Yes	146 (4.7)	28 (3.1)	118 (5.5)	
**Delivery status**				
None	1,334 (43.4)	433 (47.2)	901 (41.8)	0.006
Previous	1,742 (56.6)	485 (52.8)	1,257 (58.2)	
**Dietary folate concentration adjusted by energy (ug/1,000 kcal)**				
Low	1,010 (32.8)	426 (46.4)	584 (27.0)	< 0.001
Medium	1,061 (34.5)	311 (33.9)	750 (34.8)	
High	1,005 (32.7)	181 (19.7)	824 (38.2)	
**Region of residence**				
North	1,449 (47.1)	611 (66.6)	838 (38.8)	< 0.001
South	1,627 (52.9)	307 (33.4)	1,320 (61.2)	
**Season of enrollment**				
Spring	1,515 (49.3)	405 (44.1)	1,110 (51.4)	< 0.001
Autumn	1,561 (50.7)	513 (55.9)	1,048 (48.6)	

* Chi-square test was adopted to examine differences in plasma folate deficiency (<10.5 nmol/L vs. 10.5 nmol/L) in each categorical variable.

# Imputation methods were performed in missing values.

§ USD, United States dollar.

The prevalence of adiposity (defined by either BMI or WC) was 21.0%, of which 16.4% were general adiposity (BMI ≥ 24 kg/m^2^) and 8.8% were central adiposity (WC > 80 cm). Women with exclusively central adiposity (WC > 80 cm only), with exclusively general adiposity (BMI ≥ 24 kg/m^2^ only) and both general and central adiposity (≥24 kg/m^2^ and WC > 80 cm) accounted for 2.0, 9.7, and 6.7%, respectively. Women with abnormal WHR and WHtR accounted for 8.3 and 11.5%, respectively (Table 2). However, no significant differences in the percentage of plasma folate deficiency by education, smoking and WHR were detected.

**Table 2:** Adiposity status among Chinese women aged 18–30 years, 2005–2006^*^.

**Anthropometric**	**Total**	**Plasma folate**	**Non-plasma folate**	***P***
**indicators**		**deficiency**	**deficiency**	
	**(*n* = 3,076)**	**(*n* = 918)**	**(*n* = 2,158)**	
**BMI (kg/m** ^**2**^ **)**				
Underweight	460 (15.0)	103 (22.4)	357 (77.6)	< 0.001
Normal weight	2,110 (68.6)	603 (28.6)	1,507 (71.4)	
Overweight^†^	506 (16.4)	212 (41.9)	294 (58.1)	
**WC (cm)**				
Normal	2,806 (91.2)	802 (28.6)	2,004 (71.4)	< 0.001
Central adiposity^‡^	270 (8.8)	116 (43.0)	154 (57.0)	
**WHR**				
≤0.85	2,821 (91.7)	840 (29.8)	1,981 (70.2)	0.786
> 0.85	255 (8.3)	78 (30.6)	177 (64.9)	
**BMI and WC combined** ^§^				
Non-increased BMI and WC	460 (15.0)	103 (22.4)	357 (77.6)	< 0.001
Normal BMI and WC	2,049 (66.6)	582 (28.4)	1,467 (71.6)	
Exclusively central adiposity	61 (2.0)	21 (34.4)	40 (65.6)	
Exclusively general adiposity	299 (9.7)	117 (39.1)	182 (60.9)	
Both general and central adiposity	207 (6.7)	95 (45.9)	112 (54.1)	
WHtR				
≤0.5	2,721 (88.5)	786 (28.9)	1,935 (71.1)	0.001
>0.5	355 (11.5)	132 (37.2)	223 (62.8)	

* Chi-square test was adopted to examine differences in plasma folate deficiency (< 10.5 nmol/L vs. 10.5 nmol/L) in each categorical variable.

† Obesity group was combined with overweight group due to limited sample size (n = 82).

‡ Central adiposity was defined as WC > 80 cm.

§ Non-increased BMI and WC (BMI ≤ 18.5 kg/m^2^, WC ≤ 80 cm); Normal BMI and WC (18.5 kg/m^2^ < BMI < 24 kg/m^2^, WC ≤ 80 cm); Exclusively central adiposity (18.5 kg/m^2^ < BMI < 24 kg/m^2^, WC > 80 cm); Exclusively general adiposity (BMI ≥ 24 kg/m^2^, WC ≤ 80 cm); With both general and central adiposity (BMI ≥ 24 kg/m^2^, WC > 80 cm).

BMI, body mass index; WC, waist circumference; WHtR, waist-to-height ratio; WHR, waist-to-hip ratio.

### Association between adiposity (by BMI and WC separately) and odds for plasma folate deficiency

Table 3 shows the relationship between plasma folate deficiency and related factors. Other ethnic groups had higher odds for plasma folate deficiency than Han ethnic groups (OR =3.225, 95% CI: 2.618, 3.972). Compared to low dietary folate intake, higher dietary folate intake was associated with a lower likelihood of plasma folate deficiency [OR = 0.775 (medium) and 0.483 (high), respectively]. After adjustment for multiple potential confounders, general adiposity was associated with higher odds for plasma folate deficiency in these women than in normal weight women (OR = 1.637, 95% CI: 1.237–2.166), and central adiposity was also associated with higher odds for plasma folate deficiency than in women with normal WC (OR = 1.906, 95% CI: 1.159–3.137) (Table 3).

**Table 3:** Logistic regression of factors correlated with the odds of folate deficiency in Chinese women aged 18–30 years^‡^^#^.

**Variables (*n* = 3,076)**	***P***	**OR**	**95%CI**	
			**Lower**	**Upper**
			**bound**	**bound**
**Age (years)**				
18–24		Ref		
25–30	0.276	0.884	0.708	1.104
**Ethnicity**				
Han ethnic		Ref		
Others	< 0.001	3.225	2.618	3.972
**Occupation**				
Farmers		Ref		
Workers	<0.001	3.261	2.252	4.721
Unemployed	0.005	1.521	1.137	2.034
Others	0.026	1.502	1.050	2.149
**Income [Yuan (USD), per year]**				
< 3,000 (442.8)		Ref		
3,000–7,000 (442.8–1033.2)	0.069	1.243	0.983	1.570
≥7,000 (1033.2)	< 0.001	0.587	0.438	0.788
**Residence type**				
Rural		Ref		
Urban	0.041	0.749	0.568	0.988
**Oral contraceptive**				
No		Ref		
Yes	**0.006**	2.344	1.278	4.299
**Drinking alcohol beverage**				
No		Ref		
Yes	0.607	1.092	0.780	1.529
**Folic acid supplementation**				
No		Ref		
Yes	0.205	0.733	0.454	1.184
**Delivery status**				
None		Ref		
Yes	0.478	0.916	0.719	1.167
**BMI (kg/m** ^**2**^ **)**				
Underweight (≤18.5)	0.042	0.747	0.564	0.989
Normal weight (18.5–24)		Ref		
Overweight (≥24)	0.001	1.637	1.237	2.166
**Waist circumference (cm)**				
Normal WC (≤80)		Ref		
Central adiposity (>80)	0.011	1.906	1.159	3.137
Low		Ref		
Medium	0.018	0.775	0.628	0.957
High	< 0.001	0.483	0.382	0.611
**Region of residence**				
North		Ref		
South	<0.001	0.211	0.170	0.261
**Season of enrollment**				
Spring		Ref		
Autumn	< 0.001	1.423	1.185	1.710
**WHtR**				
≤0.5		Ref		
>0.5	0.579	0.875	0.545	1.405

‡ Adjusted for age, ethnicity, occupation, oral contraceptives, delivery status, FA supplement, alcoholic beverage drinking, season, income, BMI, WC, dietary folate intake adjusted by energy, residence type, region of residence, WHtR.

# Imputation methods were performed in missing values.

BMI, body mass index; WC, waist circumference; WHtR, waist-to-height ratio; WHR, waist-to-hip ratio; USD, United States dollar.

### Association between adiposity (by combination of BMI and WC) and odds for plasma folate deficiency

Then, we further investigated the combination of BMI and WC with the odds of plasma folate deficiency after controlling for confounders (Table 4). Compared with women with normal BMI and WC, women non-increased BMI and WC were a protective factor for plasma folate deficiency (OR = 0.754, 95% CI: 0.569–0.999), women with exclusively central adiposity had an elevated likelihood for plasma folate deficiency (OR = 2.448, 95% CI: 1.144–5.241), which was higher than women with exclusively general adiposity (OR = 1.709, 95% CI: 1.259–2.319), and women with both central and general adiposity had the highest odds for plasma folate deficiency (OR = 3.107, 95% CI: 1.819–5.307).

**Table 4:** Odd ratios and 95% confidence interval for the association of adiposity (measured by the combination of BMI and WC) with plasma folate deficiency risk among Chinese women aged 18–30 years^#^^†^^‡^.

**BMI and WC combined (*n* = 3,076)**	***P***	**OR**	**95%CI**	
			**Lower bound**	**Upper bound**
Non-increased BMI and WC	0.049	0.754	0.569	0.999
Normal BMI and WC		Ref		
Exclusively central adiposity	0.021	2.448	1.144	5.241
Exclusively general adiposity	0.001	1.709	1.259	2.319
With both general and central adiposity	< 0.001	3.107	1.819	5.307

† Adjusted for age, ethnicity, occupation, oral contraceptives, delivery status, FA supplement, alcoholic beverage drinking, season, income, dietary folate intake adjusted by energy, residence type, region of residence, WHtR.

# Imputation methods were performed in missing values.

‡ Non-increased BMI and WC (BMI ≤ 18.5 kg/m^2^, WC ≤ 80 cm); Normal BMI and WC (18.5 kg/m^2^ < BMI < 24 kg/m^2^, WC ≤ 80 cm); Exclusively central adiposity (18.5 kg/m^2^ < BMI < 24 kg/m^2^, WC > 80 cm); Exclusively general adiposity (BMI ≥ 24 kg/m^2^, WC ≤ 80 cm); With both general and central adiposity (BMI ≥ 24 kg/m^2^, WC > 80 cm).

BMI, body mass index; WC, waist circumference; WHtR, waist-to-height ratio.

## Discussion

The present study investigated the relationships between body adiposity and the likelihood of plasma folate deficiency among 3,076 women of reproductive age aged 18–30 years in China. Compared to women with normal BMI and WC, women with both general and central adiposity were 3.107 times more deficient in plasma folate. Notably, women with exclusively central adiposity in normal BMI were at higher odds for plasma folate deficiency than women with exclusively general adiposity in normal WC (2.448-vs. 1.709-fold), which supported our hypothesis. Moreover, nearly 12% of women at higher plasma folate deficiency odds would be neglected if BMI or WC were used alone.

There is an increasing trend in the prevalence of adiposity among adults aged 18–45 years in China, from 12.1% (1989) to 36.8% (2018) (29). This study revealed that more than one-fifth of Chinese women of reproductive age were adiposity when measured either by BMI, WC, WHR, or WHtR. We further distinguished subgroups of women with exclusively central adiposity, women with exclusively general adiposity and women with both general and central adiposity. In the present study, of women with normal weight, 2.0% had elevated levels of WC (>80 cm), while 9.7% of overweight/obese women had normal WC. These women would be missed if only one index was used. However, very few studies have avoided this problem by using both indexes. It has been reported that nearly two-thirds of obese cases would be undiagnosed if WC was not measured in China, which may lead to unanticipated adverse clinical outcomes (30). Previous studies tended to investigate the relationship between BMI and the odds of folate deficiency separately, so it is difficult to compare the predictive value of each directly. Since adiposity prevalence would be low-estimated due to BMI or WC used alone, combined measures of BMI and WC can help identify more women who might have potentially elevations in plasma folate deficiency risk. Thus, the results from our study strengthen the fact that both BMI and WC are screening plasma folate deficiency risk tools among women of reproductive age in China.

This study found that general adiposity correlated negatively with plasma folate and was more likely to be deficient in women with a BMI ≥ 24 kg/m^2^ than in women who had a normal BMI (BMI 18.5–23.9 kg/m^2^). Our findings are consistent with previous studies. Shen et al. (31) found that higher BMI was associated with lower serum folate levels and higher RBC folate levels among pregnant women, and Scholing et al. (32) reported that prepregnancy BMI was negatively correlated with low serum folate concentration (9). The possible mechanism for such a relationship may include urinary excretion (increased estrogen concentrations in obese individuals may increases folate utilization by its requirement for methylation *via* catechol-O-methyltransferase before urinary excretion), dilution of the blood volume at different levels in different tissues (modifications in plasma volume may affect the distribution, transportation and endocrine functions of folate) (10, 33). Another reason may be that adiposity may affect the absorption of folate by the intestinal epithelium (4). However, some studies have failed to show an association between BMI and folate levels. A cross-sectional study conducted in the USA in 2007–2010 reported that there was no relationship between RBC folate and BMI among non-pregnant reproductive women (34). This may be due to population differences and criteria of adiposity, and probably, with its limitation of not reflecting body size, indicating that obesity stratification simply by BMI may not be sufficient. Our study suggested that indicators beyond BMI need further investigation. This study revealed that women with central adiposity had a higher risk of plasma folate deficiency than women with normal WC, which indicated that central adiposity may be related to lower folate levels (35). Our findings are consistent with previous studies. A recent case-control study conducted in Poland reported that lower serum folate was independently associated with greater body weight and central adiposity in people aged 20–40 years (36). Our study further revealed that women with exclusively central adiposity were at higher risk for plasma folate deficiency than women with exclusively general adiposity (OR: 2.448 vs. 1.709), indicating that central adiposity may be more harmful for women due to its increased plasma folate deficiency risk compared to general adiposity. In addition to the similar mechanisms affecting folate levels with BMI, the additional possible mechanisms may be that abdominal adiposity is correlated with systemic oxidative stress (37), glucose metabolism (6) and DNA methylation (38). The study of Mlodzik-Czyzewska et al. (36) revealed that folate may affect body fat distribution through epigenetic mechanisms. In addition, homocysteine metabolism is carried out by enzymes that use folate as a cofactor. Previous studies revealed that the drop in folate levels leads to an increase in homocysteine, so the total homocysteine concentration among obese individuals was higher than that among their counterparts of normal weight (39). Higher homocysteine levels were correlated with abdominal fat accumulation and insulin resistance, which were negatively associated with folate (40). An increase in central adiposity may not be correlated with the change in BMI. When in the same BMI, these individuals may have more fat mass aggregation in the abdomen and less lean mass or subcutaneous adipose tissue in the arms or legs. Compared to subcutaneous adipose tissue, visceral adipose tissue has a stronger impact on metabolic syndrome and insulin resistance (41, 42). Our results underscore the need for early detection for this subgroup of individuals to prevent NTDs in their offspring.

Our study has several strengths. First, we used two indicators (BMI and WC) to measure body adiposity to comprehensively explore the separate effects of BMI and WC and the joint associations of BMI and WC. We also controlled for other central adiposity-related indicators (WHR, WHtR), which may more clearly investigate the relationship between central adiposity (measured by WC or the combination of BMI and WC) and plasma folate deficiency. Second, our study enrolled women from different areas of China with different background characteristics and lifestyle information who were representative of Chinese women. Third, we collected information on dietary intake from both the diet and supplements, which strengthened our results.

Our study is subject to several limitations. First, we focused only on short-term folate status and did not measure RBC folate. Second, this was a cross-sectional study, so we could not establish a cause-effect relationship. A further large-scale cohort study is required to explore the relationship between adiposity and blood folate levels by using more indicators.

In conclusion, among Chinese women of reproductive age aged 18–30 years, women with central adiposity were at higher risk for plasma folate deficiency than women with general adiposity. The combined use of BMI and WC appeared to be more predictive of plasma folate deficiency risk factors, especially for normal-weight women. Our results underscore the need for future public health guidelines to include the prevention and control of central adiposity, even in individuals with normal BMI.

## Data availability statement

The original contributions presented in the study are included in the article/supplementary materials, further inquiries can be directed to the corresponding author/s.

## Ethics statement

The studies involving human participants were reviewed and approved by the Institutional Review Board of Peking University Health Science Center. The patients/participants provided their written informed consent to participate in this study.

## Author contributions

JZ wrote the manuscript. JL conceptualized the manuscript, wrote, reviewed, and edited the manuscript. SX, XC, and YD contributed to the statistical analysis discussion, reviewed, and edited the manuscript. JL and LZ were involved in the study design and data collection and contributed to discussion, review, and critical revision. All authors contributed to the article and approved the submitted version.

## Funding

This work was supported by Chinese Nutrition Society-ZD Tizhi and Health Fund (CNS-ZD2020-115), the Fundamental Research Funds for the Central Universities (BMU2021YJ034), and WHO-Ministry of Health (MOH) 2004–2005 Cooperation Project (Grant Number WP/2004/CHN/RPH/3·4/001).

## Conflict of interest

The authors declare that the research was conducted in the absence of any commercial or financial relationships that could be construed as a potential conflict of interest.

## Publisher's note

All claims expressed in this article are solely those of the authors and do not necessarily represent those of their affiliated organizations, or those of the publisher, the editors and the reviewers. Any product that may be evaluated in this article, or claim that may be made by its manufacturer, is not guaranteed or endorsed by the publisher.

## References

[1] Lassi ZS Kedzior SGE Tariq W Jadoon Y Das JK Bhutta ZA. Effects of preconception care and periconception interventions on maternal nutritional status and birth outcomes in low- and middle-income countries: a systematic review. Nutrients. (2020) 12:606. 10.3390/nu12030606 32110886 PMC7146400

[2] van Gool JD Hirche H Lax H De Schaepdrijver L. Folic acid and primary prevention of neural tube defects: a review. Reprod Toxicol. (2018) 80:73–84. 10.1016/j.reprotox.2018.05.004 29777755

[3] Thomas-Valdés S Tostes MdGV Anunciação PC da Silva BP Sant'Ana HMP. Association between vitamin deficiency and metabolic disorders related to obesity. Crit Rev Food Sci Nutr. (2017) 57:3332–43. 10.1080/10408398.2015.1117413 26745150

[4] Bird JK Ronnenberg AG Choi SW Du F Mason JB Liu Z. Obesity is associated with increased red blood cell folate despite lower dietary intakes and serum concentrations. J Nutr. (2015) 145:79–86. 10.3945/jn.114.199117 25527662 PMC6619680

[5] Kimmons JE Blanck HM Tohill BC Zhang J Khan LK. Associations between body mass index and the prevalence of low micronutrient levels among US adults. Med Gen Med. (2006) 8:59. 17415336 PMC1868363

[6] Mojtabai R. Body mass index and serum folate in childbearing age women. Eur J Epidemiol. (2004) 19:1029–36. 10.1007/s10654-004-2253-z 15648596

[7] Yang Y Cai Z Zhang J. The effect of prepregnancy body mass index on maternal micronutrient status: a meta-analysis. Sci Rep. (2021) 11:18100. 10.1038/s41598-021-97635-3 34518612 PMC8437962

[8] Wiebe N Field CJ Tonelli M. A systematic review of the vitamin B12, folate and homocysteine triad across body mass index: systematic review of B12 concentrations. Obes Rev. (2018) 19:1608–18. 10.1111/obr.12724 30074676

[9] Soysal P Smith L Capar E Kalan U Arik F Isik AT. Vitamin B12 and folate deficiencies are not associated with nutritional or weight status in older adults. Exp Gerontol. (2019) 116:1–6. 10.1016/j.exger.2018.12.007 30550763

[10] Köse S Sözlü S Bölükbaşi H Ünsal N Gezmen-Karadag M. Obesity is associated with folate metabolism. Int J Vitam Nutr Res. (2020) 90:353–64. 10.1024/0300-9831/a000602 31512572

[11] Hwang S Park YM Han KD Yun JS Ko SH Ahn YB. Associations of general obesity and central obesity with the risk of hepatocellular carcinoma in a Korean population: a national population-based cohort study. Int J Cancer. (2021) 148:1144–54. 10.1002/ijc.33305 32955731

[12] Sangrós FJ Torrecilla J Giráldez-García C Carrillo L Mancera J Mur T. Association of general and abdominal obesity with hypertension, dyslipidemia and prediabetes in the PREDAPS study. Rev Española Cardiol. (2018) 71:170–7. 10.1016/j.rec.2017.04.035 28789915

[13] Mahabir S Ettinger S Johnson L Baer DJ Clevidence BA Hartman TJ. Measures of adiposity and body fat distribution in relation to serum folate levels in postmenopausal women in a feeding study. Eur J Clin Nutr. (2008) 62:644–50. 10.1038/sj.ejcn.1602771 17457338 PMC3236439

[14] Gunanti IR Marks GC Al-Mamun A Long KZ. Low serum vitamin B-12 and folate concentrations and low thiamin and riboflavin intakes are inversely associated with greater adiposity in Mexican American children. J Nutr. (2014) 144:2027–33. 10.3945/jn.114.201202 25411037

[15] Niu J Seo DC. Central obesity and hypertension in Chinese adults: a 12-year longitudinal examination. Prev Med. (2014) 62:113–8. 10.1016/j.ypmed.2014.02.012 24552844

[16] Chen Y Yang Y Jiang H Liang X Wang Y Lu W. Associations of BMI and waist circumference with all-cause mortality: a 22-Year cohort study. Obesity. (2019) 27:662–9. 10.1002/oby.22423 30861324

[17] Shen C Zhou Z Lai S Tao X Zhao D Dong W. Urban-rural-specific trend in prevalence of general and central obesity, and association with hypertension in Chinese adults, aged 18–65 years. BMC Public Health. (2019) 19:661. 10.1186/s12889-019-7018-4 31146734 PMC6543650

[18] Zhang L Wang Z Wang X Chen Z Shao L Tian Y. Prevalence of abdominal obesity in china: results from a cross-sectional study of nearly half a million participants. Obesity. (2019) 27:1898–905. 10.1002/oby.22620 31549787

[19] Bosomworth NJ. Normal-weight central obesity: unique hazard of the toxic waist. Can Fam Physician. (2019) 65:399–408. 31189627 PMC6738397

[20] O'Súilleabháin PS Sutin AR Gerstorf D. Body mass index, waist circumference, and mortality risks over 27 years of follow-up in old age. Ann Epidemiol. (2020) 46:20–3. 10.1016/j.annepidem.2020.04.008 32532369

[21] Jia X Ren M Zhang Y Ye R Zhang L Li Z. Association between tea drinking and plasma folate concentration among women aged 18–30 years in China. Public Health Nutr. (2020) 36:1–8. 10.1017/S1368980020004851 33317650 PMC11082794

[22] Yang YX. Chinese Food Composition, 2004. Beijing: Peking University Medical Press (2004).

[23] Department of Health Disease Control of Health Ministry of the People's Republic of China. The Guidelines for Prevention and Control of Overweight and Obesity in Chinese Adults. People's Medical Publishing House (2006).

[24] Zhai Y Zhao WH Chen CM. Verification of the cut-off waist circumference for defining central obesity in Chinese adults. Chin J Epidemiol. (2006) 137:S101. 10.1016/j.ijcard.2009.09.341 21163090

[25] Editing Group for Guidelines for prevention and treatment of diabetes in China. Guidelines for Prevention and Treatment of Diabetes in China. Beijing: Peking University Medical Press (2004).

[26] Zhao LC Ying LI Peng YG Zhang LF Guo M. The cut-off value of waist-to-height ratio in detecting central obesity in Chinese adult population (In Chinese). Chin Prev Med. (2012) 13:481–5.24284192

[27] O'Broin S Kelleher B. Microbiological assay on microtitre plates of folate in serum and red cells. J Clin Pathol. (1992) 45:344–7. 10.1136/jcp.45.4.344 1577973 PMC495277

[28] Hao L Zheng J chi Tian Y hua Fan D wei Li Z. Comparative study of the detection of plasma folate with microbial assay and radioimmunoassay (In Chinese). Beijing Da Xue Xue Bao Yi Xue Ban. (2004) 36:210–4. 15100746

[29] HAO L ZHANG B Wang H Wang L Jiang H Wang Z. Trends and epidemic characteristics of overweight and obesity among adults aged 18-35 in 15 provinces (autonomous regions/municipalities) of China from 1989 to 2018. J Environ Occup Med. (2022) 39:471–7. 10.11836/JEOM21386

[30] Du T Sun X Yin P Huo R Ni C Yu X. Increasing trends in central obesity among Chinese adults with normal body mass index, 1993-2009. BMC Public Health. (2013) 13:327. 10.1186/1471-2458-13-327 23575244 PMC3626835

[31] Shen M Chaudhry SH MacFarlane AJ Gaudet L Smith GN Rodger M. Serum and red-blood-cell folate demonstrate differential associations with BMI in pregnant women. Public Health Nutr. (2016) 19:2572–9. 10.1017/S1368980016000756 27087411 PMC10270987

[32] Scholing JM Olthof MR Jonker FA Vrijkotte TG. Association between pre-pregnancy weight status and maternal micronutrient status in early pregnancy. Public Health Nutr. (2018) 21:2046–55. 10.1017/S1368980018000459 29560851 PMC10261015

[33] Nakazato M Maeda T Takamura N Wada M Yamasaki H Johnston KE. Relation of body mass index to blood folate and total homocysteine concentrations in Japanese adults. Eur J Nutr. (2011) 50:581–5. 10.1007/s00394-010-0165-0 21221977

[34] Manandhar M Beydoun H Kancherla V. Association between body mass index and folate insufficiency indicative of neural tube defects risk among nonpregnant women of childbearing age in the United States, NHANES, 2007–2010. Birth Defects Res. (2020) 112:490–502. 10.1002/bdr2.1658 32052935

[35] Song P Li X Bu Y Ding S Zhai D Wang E. Temporal trends in normal weight central obesity and its associations with cardiometabolic risk among Chinese adults. Sci Rep. (2019) 9:5411. 10.1038/s41598-019-41986-5 30931996 PMC6443661

[36] Mlodzik-Czyzewska MA Malinowska AM Chmurzynska A. Low folate intake and serum levels are associated with higher body mass index and abdominal fat accumulation: a case control study. Nutr J. (2020) 19:53. 10.1186/s12937-020-00572-6 32498709 PMC7273685

[37] Kelli HM Corrigan FE Heinl RE Dhindsa DS Hammadah M Samman-Tahhan A. Relation of changes in body fat distribution to oxidative stress. Am J Cardiol. (2017) 120:2289–93. 10.1016/j.amjcard.2017.08.053 29102347 PMC5810365

[38] Frank AP de Souza Santos R Palmer BF Clegg DJ. Determinants of body fat distribution in humans may provide insight about obesity-related health risks. J Lipid Res. (2019) 60:1710–9. 10.1194/jlr.R086975 30097511 PMC6795075

[39] Mehmetoglu I Yerlikaya FH Kurban S Polat H. Plasma ω-3 fatty acid levels negatively and ω-6 fatty acid levels positively associated with other cardiovascular risk factors including homocysteine in severe obese subjects. Asia Pac J Clin Nutr. (2012) 21:519–25. 23017310

[40] Vayá A Rivera L Hernández-Mijares A de la Fuente M Solá E Romagnoli M. Homocysteine levels in morbidly obese patients: its association with waist circumference and insulin resistance. Clin Hemorheol Microcirc. (2012) 52:49–56. 10.3233/CH-2012-1544 22460264

[41] Chen S Chen Y Liu X Li M Wu B Li Y. Insulin resistance and metabolic syndrome in normal-weight individuals. Endocrine. (2014) 46:496–504. 10.1007/s12020-013-0079-8 24190050

[42] da Silva RP Kelly KB Al Rajabi A Jacobs RL. Novel insights on interactions between folate and lipid metabolism. Biofactors. (2014) 40:277–83. 10.1002/biof.1154 24353111 PMC4153959

